# The impact of abusive supervision on employee counterproductive work behavior: a moderated mediation analysis

**DOI:** 10.3389/fpsyg.2025.1455658

**Published:** 2025-07-04

**Authors:** Jingli Li, Guangnuo Xu

**Affiliations:** School of Management, Henan University of Technology, Zhengzhou, China

**Keywords:** abusive supervision, emotional exhaustion, ingratiation behavior, core self-evaluation, counterproductive work behavior

## Abstract

**Introduction:**

This study explores how abusive supervision impacts employee counterproductive work behavior (CWB), highlighting the mediating roles of emotional exhaustion and ingratiation behavior, and the moderating role of core self-evaluation.

**Methods:**

Drawing on self-regulation theory and resource conservation theory, the paper tests a moderated mediation model using a three-wave survey of 198 employees.

**Result and discussion:**

Results indicate that abusive supervision directly increases CWB, with emotional exhaustion serving as a positive mediator and ingratiation behavior serving as a negative mediator. Core self-evaluation moderates both mediation paths. These findings provide new insights into the dual psychological mechanisms underlying workplace deviance and suggest practical strategies for mitigating abusive leadership.

## 1 Introduction

Abusive supervision (AS)—defined as a supervisor’s sustained display of hostile verbal and non-verbal behavior excluding physical contact (Tepper, 2000; Hameed et al., 2021; Asim et al., 2023)—has emerged as a significant focus in organizational behavior research. AS not only affects individual psychological well-being but also leads to broader organizational dysfunction. Counterproductive work behavior (CWB), such as workplace deviance or withdrawal, has often been identified as a behavioral response to abusive supervisory treatment.

Despite extensive literature linking AS with CWB, the underlying psychological mechanisms remain inadequately understood. This study introduces a dual-pathway model incorporating both emotional exhaustion (EE) and ingratiation behavior (IB) as mediators. Drawing on Conservation of Resources (COR) theory (Hobfoll, 1989) and Self-Regulation Theory (Carver et al., 1999; Saleem et al., 2024), we propose that AS depletes employees’ emotional and self-regulatory resources, thereby influencing their emotional state and interpersonal strategy.

Moreover, individual differences may buffer or amplify the effects of AS. Core self-evaluation (CSE), a higher-order personality trait reflecting individuals’ self-worth and competence (Judge et al., 2003), is explored as a moderator of the AS and emotional state as well as interpersonal strategy relationships.

By investigating these mediating and moderating mechanisms, our study extends theoretical understanding of how abusive leadership shapes employee behavior and offers new directions for intervention and leadership training.

The primary purpose of this article is to explore the ways in which employees engage in counterproductive work behavior (CWB) as a response to AS. CWB, which include actions such as theft, sabotage, and workplace deviance, are posited to be direct reactions to the stress caused by abusive supervision (Cao et al., 2023). By investigating these behaviors, the study seeks to highlight the immediate, tangible consequences of toxic leadership on organizational efficacy and employee well-being.

A secondary objective of this research is to verify the mediation effects of emotional exhaustion (EE) and ingratiation behavior (IB) on the relationship between AS and CWB. EE, a chronic state of physical and emotional depletion resulting from excessive workplace demands and stress (Wright and Cropanzano, 1998), and ingratiation behavior (Vonk, 2002), characterized by a deep-seated mistrust and negative attitude towards the organization, are examined as potential mediators. The study aims to elucidate how these psychological states translate the experience of abusive supervision into detrimental or beneficial behaviors, thereby providing a deeper psychological understanding of the process.

Finally, the article aims to contribute to the existing body of knowledge by providing empirical evidence on the psychological mechanisms and individual differences that underlie the impact of AS. It offers insights for practitioners on how to mitigate the negative consequences of such supervision through targeted interventions, such as promoting CSE and addressing EE and IB. The findings have the potential to inform policies and training programs aimed at fostering a healthier, more supportive organizational environment, ultimately enhancing employee well-being and organizational performance.

In summary, this article endeavors to provide a comprehensive analysis of AS, emphasizing its significant organizational outcomes and the psychological processes involved. Through a detailed examination of CWB, the mediation effects of EE and IB, and the moderation role of CSE, the study offers valuable contributions to both research and practical management.

## 2 Theoretical foundation and hypothesis development

### 2.1 Abusive supervision and emotional exhaustion

Abusive supervision (AS) refers to supervisors’ sustained hostile verbal and nonverbal behaviors toward subordinates, excluding physical aggression (Tepper, 2000; Fischer et al., 2021; Liu et al., 2025). These behaviors can include frequent criticism, public humiliation, belittling remarks, and unreasonable task assignments, significantly undermining subordinates’ self-worth and psychological well-being. Emotional exhaustion (EE), a critical dimension of burnout, manifests as feelings of being emotionally overextended and depleted of emotional resources, leading to decreased work engagement and diminished performance (Maslach and Jackson, 1981).

Conservation of Resources Theory (COR) posits that individuals strive continuously to acquire, retain, and protect valuable resources, such as emotional stability, self-esteem, and psychological well-being. When these resources are threatened, depleted, or lost due to persistent stressors, individuals experience strain and negative outcomes (Hobfoll, 1989). Abusive supervision acts precisely as such a persistent workplace stressor, continuously depleting employees’ psychological and emotional resources and thereby increasing their vulnerability to emotional exhaustion (Al-Hawari et al., 2020; Lee et al., 2022; Landmann et al., 2024). Moreover, prolonged exposure to abusive supervision can create an environment characterized by ongoing fear and anxiety, further accelerating the depletion of emotional resources.

Recent empirical evidence underscores the robustness of this relationship. Wu et al. (2023), for example, demonstrated that abusive supervision consistently predicted heightened emotional exhaustion among employees, particularly when subordinates engaged in maladaptive emotional regulation strategies like expressive suppression rather than cognitive reappraisal. Consequently, continuous exposure to abusive supervisory behaviors amplifies emotional distress and significantly elevates emotional exhaustion.

Hypothesis 1: Abusive supervision is positively related to emotional exhaustion.

### 2.2 Abusive supervision and ingratiation behavior

Ingratiation behavior (IB) involves proactive strategies aimed at enhancing one’s attractiveness or likability to others, typically through flattery, praise, or other forms of impression management (Vonk, 2002). Employees engage in ingratiation behaviors as adaptive responses to unfavorable or threatening situations, intending to secure social and professional advantages, reduce interpersonal conflict, and alleviate negative outcomes.

Self-Determination Theory suggests that abusive supervision significantly undermines subordinates’ fundamental psychological needs for autonomy, competence, and relatedness, prompting them to seek alternative strategies to restore psychological resources and reduce perceived threats (Breevaart et al., 2022; Hobfoll, 1989; Mackey et al., 2017). Consequently, employees facing abusive supervision may resort to ingratiation behaviors as a strategic coping mechanism aimed at mitigating negative supervisory evaluations, reducing hostility, and enhancing their sense of security and acceptance within the organization.

Recent research highlights ingratiation behavior as a tactical response to abusive supervision. Khan et al. (2023) found that employees experiencing sustained abusive supervision were more inclined to adopt ingratiation tactics to alleviate workplace stress and frustration. Similarly, Liu et al. (2023) suggested that ingratiation serves as a resource-restoration strategy, enabling employees to rebuild damaged relationships with supervisors and regain lost psychological resources. Such behaviors are not simply attempts at superficial impression management; rather, they represent targeted adaptive mechanisms aimed at reducing emotional distress and improving interpersonal dynamics.

Hypothesis 2: Abusive supervision is positively related to ingratiation behavior.

### 2.3 Abusive supervision and counterproductive work behavior

Counterproductive work behavior (CWB) encompasses intentional acts by employees intended to harm organizational interests, such as theft, sabotage, aggression, and intentional inefficiency (Cao et al., 2023; Fan et al., 2023). Abusive supervision creates a toxic workplace environment, significantly undermining employee morale, increasing workplace stress, and weakening overall organizational effectiveness (Tepper, 2007; Yu et al., 2023).

From a retaliatory perspective, employees subjected to abusive supervision may perceive significant violations of organizational justice and fairness, prompting negative reciprocity in the form of CWBs. Recent research has robustly confirmed the linkage between abusive supervision and increased employee deviance. For instance, Zhang et al. (2023) demonstrated through meta-analytic methods that abusive supervision consistently predicts various CWBs, such as sabotage, theft, and interpersonal aggression. Furthermore, perceived organizational injustice significantly mediates the relationship between abusive supervision and CWBs, highlighting the critical role of fairness perceptions in employees’ retaliatory actions (Wang et al., 2022).

The ongoing experience of abusive supervisory behaviors also fosters feelings of helplessness and resentment, motivating employees to engage in CWBs as a coping mechanism or as a form of indirect revenge against supervisors and the organization that implicitly tolerates abusive leadership.

Hypothesis 3: Abusive supervision is positively related to counterproductive work behavior.

### 2.4 Emotional exhaustion and counterproductive work behavior

Social Exchange Theory posits that employee behavior often reflects reciprocal exchanges with the organization and supervisors, based on perceived fairness, support, and obligation fulfillment (Li and Janmaat, 2023). Emotionally exhausted employees frequently perceive diminished organizational support and experience lower psychological attachment to organizational values and goals. Consequently, these employees are more inclined to reciprocate negatively through CWBs, including absenteeism, tardiness, reduced productivity, sabotage, or interpersonal hostility (Aliza et al., 2022; Chen et al., 2022).

Recent empirical evidence confirms the significant predictive role of emotional exhaustion in promoting CWBs. Li et al. (2023) emphasized that emotionally exhausted employees exhibit increased counterproductive behaviors as a form of emotional release or coping strategy to manage their heightened psychological distress. Similarly, Chen et al. (2020) provided additional support for this link, demonstrating that emotional exhaustion predicts higher engagement in deviant behaviors as employees attempt to restore emotional equilibrium and reduce workplace pressures.

Hypothesis 4: Emotional exhaustion is positively related to counterproductive work behavior.

Hypothesis 5: Emotional exhaustion mediates the relationship between abusive supervision and counterproductive work behavior.

### 2.5 Ingratiation behavior and counterproductive work behavior

Ingratiation behavior (IB) refers to strategic interpersonal actions aimed at gaining favor, approval, or likability from others, typically supervisors or influential organizational members. Common forms of ingratiation include flattery, opinion conformity, and favor-rendering behaviors, primarily utilized to build or preserve positive images and social relationships (Jones and Pittman, 1982; Bolino et al., 2016; Khan et al., 2022). Employees often adopt ingratiation to navigate organizational politics, enhance career opportunities, or mitigate unfavorable supervisory evaluations (Kim et al., 2022).

Impression management theory suggests that ingratiation behaviors enable individuals to proactively influence perceptions held by significant organizational actors. Specifically, ingratiation can serve as an assertive impression management strategy to construct or maintain a favorable self-image, reduce interpersonal conflicts, and enhance career-related outcomes (Ferris et al., 2007). Thus, employees who frequently engage in ingratiation behaviors may become highly vigilant about safeguarding the positive impressions they have cultivated.

Recent empirical studies further clarify this dynamic by showing that ingratiation significantly affects employees’ decisions regarding behaviors that could damage their meticulously cultivated organizational reputations. Zheng et al. (2022) demonstrated that employees who consistently engaged in ingratiation behaviors tended to avoid counterproductive work behaviors (CWBs), as engaging in deviant acts would substantially undermine their constructed favorable image. Such individuals are acutely aware of the negative consequences associated with actions perceived as harmful or disruptive to organizational interests.

Moreover, recent research by Liu et al. (2023) revealed a negative association between ingratiation behavior and CWBs. This finding underscores that ingratiating employees strategically manage their workplace behavior to ensure their organizational image remains intact and favorable, avoiding behaviors that would conflict with the image they aim to portray. Employees who proactively manage their impressions and interpersonal relationships perceive CWBs as incompatible with their strategic self-presentation goals, thus significantly reducing their propensity toward deviant organizational behaviors.

Therefore, given ingratiation behavior’s protective effects on individuals’ social and professional standing, we propose that ingratiation behavior is inversely associated with engagement in CWBs:

Hypothesis 6: Ingratiation behavior is negatively related to counterproductive work behavior.

Furthermore, ingratiation can act as a mediating mechanism between abusive supervision and counterproductive work behavior. Abusive supervision prompts employees to adopt ingratiation behaviors as protective strategies aimed at minimizing further negative repercussions or reestablishing their damaged relationships with supervisors (Liu et al., 2023; Shi et al., 2025; Zhao et al., 2022). This strategic response can, in turn, suppress CWBs by motivating employees to maintain consistency in their positive self-presentation and by promoting adherence to organizational norms to avoid damaging their rebuilt relationships. Thus, we propose:

Hypothesis 7: Ingratiation behavior mediates the relationship between abusive supervision and counterproductive work behavior.

### 2.6 The moderating effect of core self-evaluation in the relationship between abusive supervision and emotional exhaustion

Core self-evaluation (CSE) refers to an individual’s fundamental appraisals of their self-worth, competence, emotional stability, and locus of control (Bono and Judge, 2003; Judge et al., 2003). Employees high in CSE generally perceive themselves as capable, emotionally stable, and in control of their life circumstances, displaying greater resilience and psychological robustness when facing workplace stressors and adverse conditions (Zhang et al., 2022).

Self-regulation theory posits that individuals high in CSE possess more adaptive cognitive appraisal and emotional regulation strategies that allow them to effectively manage stressors and maintain emotional stability (Carver et al., 1999). Recent empirical evidence supports the moderating effect of CSE on the relationship between abusive supervision and emotional exhaustion. Wu et al. (2023), for instance, found that individuals with high CSE levels were better equipped to cope with abusive supervisory behaviors, demonstrating reduced emotional distress compared to individuals with lower CSE. Similarly, Lee et al. (2022) revealed that psychological empowerment—closely related to core self-evaluation—buffers the adverse impact of abusive supervision on emotional exhaustion, suggesting that individuals with stronger internal resources and self-beliefs experience significantly less emotional strain in adverse supervisory contexts.

Given this theoretical and empirical foundation, it can be logically argued that CSE serves as a critical psychological resource that mitigates the emotional exhaustion caused by abusive supervision. Employees high in CSE maintain positive perceptions of their own abilities and emotional resilience, thus significantly reducing the negative emotional and psychological effects of abusive leadership. Therefore, we hypothesize:

Hypothesis 8: Core self-evaluation negatively moderates the relationship between abusive supervision and emotional exhaustion, such that this positive relationship is weaker among employees with higher core self-evaluation.

### 2.7 The moderating effect of core self-evaluation in the relationship between abusive supervision and ingratiation behavior

Ingratiation behaviors are typically employed as proactive strategies to manage impressions and minimize negative social evaluations, particularly under stressful or threatening circumstances such as abusive supervision. Core self-evaluation, reflecting an individual’s overall confidence in their abilities and value, substantially influences how effectively employees respond to interpersonal stressors and workplace adversity (Judge et al., 2003; Kammeyer-Mueller et al., 2009; Zhang et al., 2022).

According to social cognitive theory, individuals with high CSE demonstrate greater self-efficacy and adaptability, empowering them to strategically employ impression management tactics like ingratiation more effectively under adverse conditions. Recent empirical evidence supports this proposition, suggesting that high CSE employees are more proactive and skillful in utilizing ingratiation as a strategic response to abusive supervision, facilitating favorable outcomes despite stressful conditions (Yu et al., 2022). For instance, high-CSE employees facing abusive supervisors are likely to deploy ingratiation behaviors tactically to reduce interpersonal tensions, strategically manage their supervisors’ perceptions, and maintain their organizational standing and relationships.

Consequently, individuals with high core self-evaluation are more effective at recognizing opportunities to use ingratiation behaviors strategically, enhancing their adaptability and reducing potential damage from abusive supervisory practices. They use ingratiation proactively to protect themselves from further supervisory hostility, leveraging their interpersonal and emotional skills effectively to manage adverse social dynamics. Thus, we propose:

Hypothesis 9: Core self-evaluation positively moderates the relationship between abusive supervision and ingratiation behavior, such that this positive relationship is stronger among employees with higher core self-evaluation.

**Figure 1:**
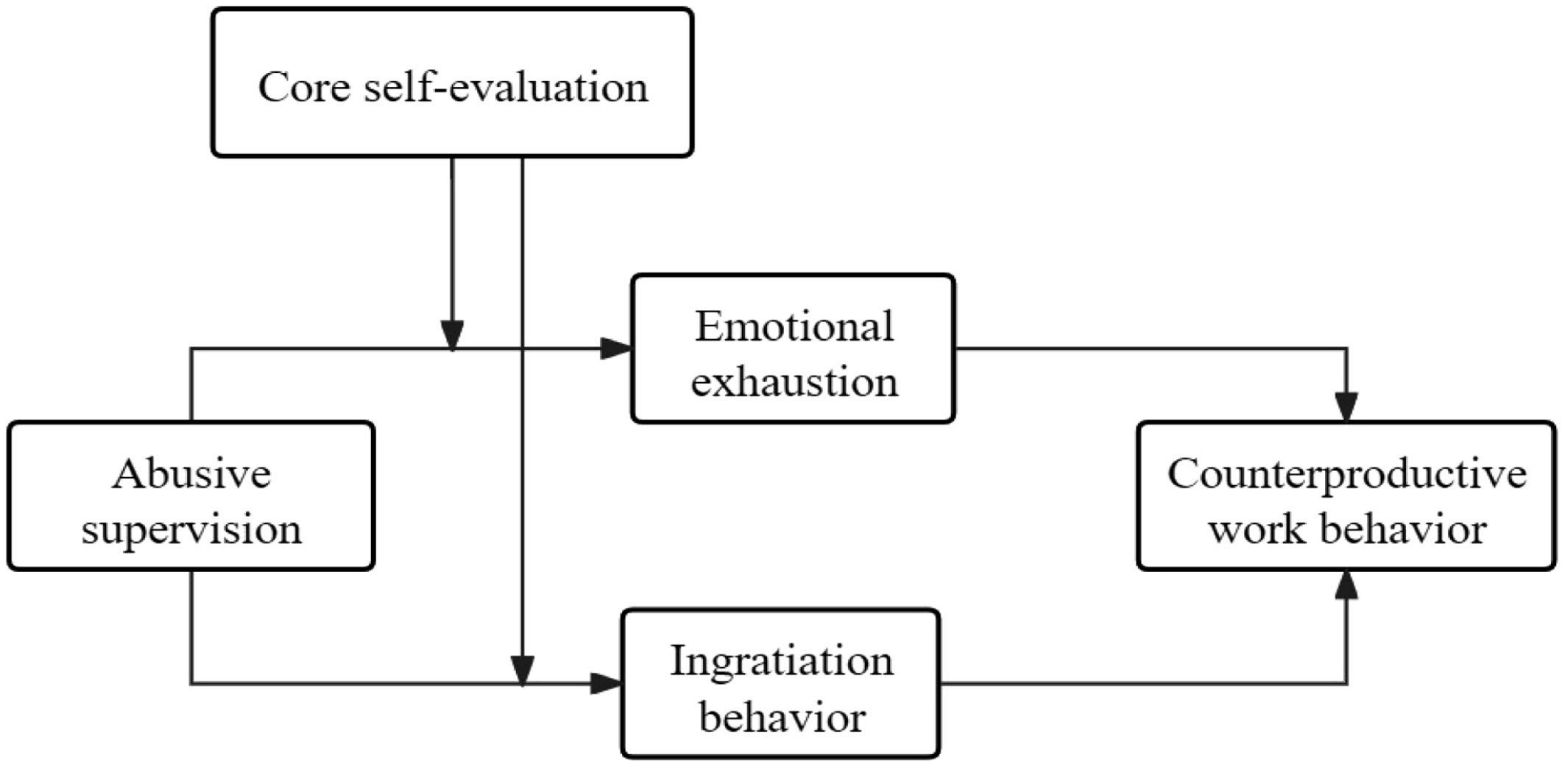
Hypothesized model.

According to the above hypotheses, the theoretical model was built in Figure 1.

## 3 Research design

### 3.1 Sample and procedure

The study was conducted in eight companies located in Henan, Jiangsu, and Beijing, covering a mix of state-owned, private, joint-venture, and foreign-invested enterprises. A three-wave survey design was adopted to reduce common method bias, with data collected through both online and paper-based formats.

At Time 1, participants (*N* = 350) provided demographic information and completed measures of abusive supervision and core self-evaluation. At Time 2, emotional exhaustion and ingratiation behavior were measured. At Time 3, participants reported their counterproductive work behavior. After matching across the three time points and eliminating incomplete responses, 198 valid samples were retained (response rate: 56.6%).

We used convenience and snowball sampling techniques due to the sensitivity of the topic and to ensure diversity in participant demographics. Participants included both frontline and administrative staff from diverse sectors.

In this survey, demographic characteristics of participants were examined across several categories: gender, age, years of work experience, education level, and company type. Regarding gender distribution, 37.9% (75) of respondents were male, while 62.1% (123) were female. The majority of participants fell within the age group of 20–29 years (67.2%), followed by those aged 30–39 years (18.2%), and a smaller proportion in other age brackets. In terms of work experience, nearly half of the respondents (49.5%) reported having less than 1 year of experience, with smaller percentages having 1–3 years (17.2%), 3–5 years (12.1%), 5–8 years (11.1%), 8–10 years (4.0%), and 10 years or more (6.1%) of experience. Regarding educational attainment, most respondents held a bachelor’s degree (69.2%), followed by those with a master’s degree (17.7%), and a smaller percentage with either a diploma or below (10.6%) or a doctorate degree (2.5%). Finally, participants were categorized based on the type of company they worked for, with the largest proportion employed in private enterprises (42.9%), followed by co-invested enterprises (28.8%), state-owned enterprises (20.2%), and foreign-invested enterprises (8.1%).

To assess the adequacy of the sample size, we conducted a post-hoc power analysis using G*Power 3.1 software. Assuming a medium effect size (*f*^2^ = 0.15), *α* = 0.05, and 4 predictors in the regression model, a sample size of 198 yields a statistical power of 0.87, exceeding the conventional threshold of 0.80. This indicates sufficient power to detect moderate effects in the proposed moderated mediation model.

### 3.2 Measures

#### 3.2.1 Abusive supervision

A 15-item scale created by Tepper (2000) was employed to assess abusive supervision, which has previously been validated in a Chinese context (such as Lyu et al., 2016). An example item from this scale is: “My supervisor ridicules me.” Respondents rated their agreement on a scale ranging from 1 (“strongly disagree”) to 5 (“strongly agree”). The internal consistency reliability of the scale, measured by Cronbach’s alpha, was 0.95.

#### 3.2.2 Emotional exhaustion

The measure of emotional exhaustion utilized in this study consisted of nine items (*α* = 0.93), adapted from the work of Maslach and Jackson (1981), and previously validated in Chinese research contexts (Chen et al., 2020). Example items included statements such as “I feel emotionally drained from my work” and “I feel used up at the end of the workday.”

#### 3.2.3 Ingratiation behavior

A nine-item scale was utilized to assess ingratiation behavior (α = 0.89), encompassing inquiries about three distinct types of ingratiation behaviors delineated in the social influence literature: conformity to opinions, enhancement of others, and rendering of favors. These questions were designed to capture respondents’ interactions with supervisors. The scale items were adapted from measures formulated by Westphal and Stern (2007). Example item is “In talking to your supervisor, to what extent do you express agreement with his/her viewpoint on a strategic issue, even when you do not completely share his/her opinion?”

#### 3.2.4 Core self-evaluation

Core self-evaluation was assessed using a 10-item scale adapted from Judge et al. (2003), and its validity has been verified in different context (Tang et al., 2023). Participants rated their level of agreement with each statement, such as “I determine what will happen in my life.” The scale demonstrated a high level of internal consistency, with a coefficient alpha of 0.84.

#### 3.2.5 Counterproductive work behavior

The scale developed by Yang and Diefendorff (2009), consisting of 10 items, was adopted. Example items include “Taking breaks beyond permissible limits.” In this study, Cronbach’s α value for this scale was 0.94.”

#### 3.2.6 Control variables

According to the previous research, gender, age, tenure and education level impact on counterproductive work behavior (Martinko et al., 2013). These variables were controlled.

### 3.3 Results

The statistical analyses were conducted by AMOS and SPSS software. Amos was used for the confirmatory factor analysis, and SPSS was used for the descriptive statistics, mediation and moderation effect analysis.

#### 3.3.1 Confirmatory factor analyses

AMOS was used to conduct the CFA for the analysis of discriminant validity of the variables in this study, including abusive supervision, emotional exhaustion, ingratiation behavior, core self-evaluation and counterproductive work behavior. Among all the factor models, the model fit of five-factor model is best compared to other models ( $\chi^{2}$ /df = 1.78, CFI = 0.931, TLI = 0.917, RMSEA = 0.063, SRMR = 0.071).

#### 3.3.2 Common method bias

To mitigate common method bias, we gathered data at two different time points and implemented rigorous procedural measures throughout our study. We assessed common method bias using Harman’s single-factor test, revealing that the largest single factor explained 17% of the variance, below the 40% threshold, indicating the absence of significant common method bias (Podsakoff et al., 2003).

#### 3.3.3 Descriptive statistics

The descriptive statistics of the variables as well as the correlations of variables were shown in Table 1. CWB was positively related to AS, EE, and negatively related to IB and CSE, which paved the way for further study.

**Table 1:** Descriptive statistics.

Variables	*M*	SD	Gender	Age	Education	Tenure	AS	EE	IB	CSE	CWB
1. Gender	–	–	—								
2. Age	–	–	−0.192**	—							
3. Education	–	–	−0.153*	0.211**	—						
4. Tenure	2.210	1.540	−0.354**	0.693**	0.097	—					
5. AS	2.220	0.870	−0.061	0.044	0.030	0.006	—				
6. EE	2.950	0.840	0.124	0.025	−0.150*	−0.049	0.498**	—			
7. IB	3.180	0.760	0.067	0.096	0.174*	0.037	0.181*	0.157*	—		
8. CSE	3.490	0.640	−0.023	−0.047	0.233**	−0.018	−0.543**	−0.527**	0.010	—	
9. CWB	2.170	0.880	−0.228**	0.005	−0.048	0.087	0.570**	0.430**	−0.152*	−0.506**	—

*N* = 198; M, mean; SD, standard deviation. *p < 0.05, **p < 0.01.

#### 3.3.4 Regression analysis

SPSS was used to test the hypotheses, and the result was shown in Tables 2–5. In Table 2, the regression analysis for Model 1, with emotional exhaustion as the dependent variable, yielded significant findings for several independent variables. Gender showed a positive relationship (*β* = 0.23, *p* = 0.05), indicating that being female is associated with higher emotional exhaustion, which was possibly caused by the work–family conflict for them. Education was negatively associated (*β* = −0.23, *p* = 0.01), suggesting that higher education levels correspond to lower emotional exhaustion. Abusive supervision exhibited a strong positive relationship (*β* = 0.49, *p* < 0.001), indicating that higher levels of abusive supervision are linked with increased emotional exhaustion, thus Hypothesis 1 was supported. The overall model was significant (*F* = 13.74, *R*^2^ = 0.30), indicating that the predictors collectively explain 30% of the variance in emotional exhaustion.

**Table 2:** Regression result.

Independent variable	Dependent variable					
	Emotional exhaustion		Ingratiation behavior		Counterproductive work behavior	
	Model 1		Model 2		Model 3	
	*β*	*p*	*β*	*p*	*β*	*p*
Gender	0.230	0.050	0.190	0.100	−0.350	0.000
Age	0.130	0.240	0.070	0.520	−0.160	0.110
Education	−0.230	0.010	0.220	0.020	0.010	0.910
Company type	0.050	0.380	−0.030	0.580	0.010	0.790
Tenure	−0.040	0.380	0.020	0.770	0.070	0.140
Abusive supervision	0.490	0.000	0.160	0.010	0.480	0.000
Emotional exhaustion					0.280	0.000
Ingratiation behavior					−0.300	0.000
*F*	13.740		2.730		21.780	
*R*^2^	0.300		0.280		0.480	

**Table 3:** Mediation effect result.

Type of effect		Effect	SE	95%LLCI	95%ULCI
Direct	AS→CWB	0.450	0.070	0.319	0.577
Indirect	AS→EE → CWB	0.120	0.050	0.040	0.213
Indirect	AS→IB → CWB	−0.040	0.020	−0.988	−0.007

**Table 4:** Moderation effect result.

Conditional effect of AS on EE at values of moderator (CSE)						
CSE	Effect	se	*t*	*p*	LLCI	ULCI
2.854	0.364	0.076	4.809	0.000	0.215	0.513
3.492	0.292	0.071	4.108	0.000	0.152	0.432
4.130	0.220	0.101	2.176	0.031	0.021	0.418

Conditional effect of AS on IB at values of moderator (CSE)						
CSE	Effect	se	*t*	*p*	LLCI	ULCI
2.854	0.089	0.080	1.117	0.266	−0.069	0.247
3.492	0.289	0.075	3.843	0.000	0.141	0.437
4.130	0.488	0.107	4.572	0.000	0.278	0.699

**Table 5:** Index of moderated mediation effect.

Mediator				
Index	Effect	SE(Boot)	BootLLCI	BootULCI
EE	−0.031	0.026	−0.092	0.015
IB	−0.093	0.041	−0.180	−0.022

In Model 2, with ingratiation behavior as the dependent variable, gender did not reach conventional levels of statistical significance (*β* = 0.19, *p* = 0.10), suggesting a marginal effect. Education (*β* = 0.22, *p* = 0.02) was positively associated, while abusive supervision (*β* = 0.16, *p* = 0.01) also showed a positive relationship, thus Hypothesis 2 was supported. The model was significant (*F* = 2.73, *R*^2^ = 0.28), indicating that the predictors explain 28% of the variance in ingratiation behavior.

Model 3 examined counterproductive work behavior as the dependent variable. Gender demonstrated a significant negative relationship (*β* = −0.35, *p* < 0.001), indicating that being female is associated with lower levels of counterproductive work behavior. Age and tenure did not show significant relationships. Abusive supervision (*β* = 0.48, *p* < 0.001) was strongly positively associated with counterproductive work behavior, thus Hypothesis 3 was supported. Emotional exhaustion was found positively related to counterproductive work behavior (*β* = 0.28, *p* < 0.001), thus Hypothesis 4 was supported. And ingratiation behavior was proved negatively connected with counterproductive work behavior (*β* = −0.30, *p* < 0.001), thus Hypothesis 6 was supported. The model was highly significant (*F* = 21.78, *R*^2^ = 0.48), indicating that the predictors collectively explain 48% of the variance in counterproductive work behavior.

Table 3 demonstrated the mediation effect results. The direct effect of abusive supervision on counterproductive work behavior was strong (*β* = 0.45, SE = 0.07, 95% CI [0.3194, 0.5773]), indicating that abusive supervision independently contributes to higher levels of CWB. Abusive supervision also indirectly influences CWB through its impact on emotional exhaustion (EE). The indirect effect via EE was found to be significant (*β* = 0.12, SE = 0.05, 95% CI [0.0404, 0.2134]), suggesting that abusive supervision increases CWB partially through increasing EE among employees, which supported Hypothesis 5.

However, the indirect effect of abusive supervision on CWB through ingratiation behavior (IB) was negative and significant (*β* = −0.04, SE = 0.02, 95% CI [−0.0988, −0.007]), suggesting that higher levels of ingratiation behavior attenuate the relationship between abusive supervision and CWB, which supported Hypothesis 7.

The results presented in Table 4 indicate significant moderation effects of core self-evaluation (CSE) on the relationship between abusive supervision (AS) and both emotional exhaustion (EE) and ingratiatory behavior (IB).

For EE, the conditional effects of AS at different levels of CSE are as follows. At CSE = 2.8542, the conditional effect of AS on EE is significant (effect = 0.3638, SE = 0.0756, t = 4.8094, *p* < 0.001, 95% CI [0.2146, 0.513]). At CSE = 3.4919, the conditional effect remains significant (effect = 0.2917, SE = 0.071, t = 4.1077, *p* < 0.001, 95% CI [0.1516, 0.4317]). At CSE = 4.1296, the conditional effect is still present but weaker (effect = 0.2195, SE = 0.1009, t = 2.1763, *p* = 0.0308, 95% CI [0.0206, 0.4185]), which verified the Hypothesis 8.

These results suggest that higher levels of CSE attenuate the positive relationship between AS and EE. As CSE increases, the effect of AS on EE decreases, indicating that individuals with stronger CSE may be more resilient to the detrimental effects of as on EE.

For ingratiatory behavior (IB), the conditional effects are as follows. At CSE = 2.8542, the conditional effect of AS on IB is not significant (effect = 0.0894, SE = 0.08, *t* = 1.1166, *p* = 0.2656, 95% CI [−0.0685, 0.2473]). At CSE = 3.4919, the conditional effect becomes significant (effect = 0.2887, SE = 0.0751, t = 3.8428, *p* = 0.0002, 95% CI [0.1405, 0.4369]). At CSE = 4.1296, the conditional effect remains significant (effect = 0.4881, SE = 0.1067, *t* = 4.5725, p < 0.001, 95% CI [0.2775, 0.6986]). Thus, Hypothesis 9 was partly supported.

These findings suggest that higher levels of CSE enhance the positive relationship between AS and IB. Individuals with stronger CSE may engage in more IB in response to AS, possibly as a coping mechanism or strategy to mitigate negative consequences or gain favor.

In summary, CSE plays a significant role in moderating the effects of AS on employees’ EE and IB. Higher levels of CSE buffer against EE but exacerbate ingratiatory behavior in response to abusive supervision. These findings underscore the complex interplay between personal cognitive characteristics (such as CSE) and the outcomes of AS in organizational settings. Future research could explore additional factors that may further influence these relationships, providing a more comprehensive understanding of how to mitigate the negative impacts of AS in the workplace.

Table 5 demonstrated the index of moderated mediation effect. In the pathway of AS→IB → CWB, the value of moderated mediation effect was −0.09, with p < 0.001, 95% CI [−0.1798, −0.022].

## 4 Discussion

This study examined the impact of abusive supervision on employee counterproductive work behavior (CWB) and uncovered the dual mediating roles of emotional exhaustion (EE) and ingratiation behavior (IB), as well as the moderating effect of core self-evaluation (CSE). Grounded in resource conservation theory and self-regulation theory, we provide novel insights into how employees react emotionally and behaviorally to abusive leadership, and how personal resources shape these reactions.

We found that EE positively mediates the relationship between AS and CWB, aligning with previous studies emphasizing the destructive nature of emotional depletion (Chi and Liang, 2013; Chen et al., 2020). In contrast, IB acted as a negative mediator, suggesting that ingratiation can serve as a strategic coping response, which in turn reduces deviant behavior—a relatively underexplored finding in prior research. The moderating role of CSE further revealed that individuals with higher self-evaluation are more resilient to emotional harm, yet more likely to strategically engage in IB under AS. These findings offer a nuanced perspective on adaptive versus maladaptive responses to supervisory mistreatment.

Furthermore, CSE was identified as a significant moderator in these relationships. Higher levels of CSE attenuate the positive relationship between AS and EE. This implies that employees with stronger CSE are more resilient to EE caused by AS. Conversely, CSE intensified the relationship between AS and IB, indicating that individuals with higher CSE might use IB more effectively to cope with AS.

### 4.1 Theoretical implications

This study contributes to several theoretical areas within organizational behavior and psychology. Firstly, it extends the understanding of abusive supervision by highlighting its differential impacts on employee behaviors through emotional and interpersonal mechanisms. The mediating roles of emotional exhaustion and ingratiation underscore the importance of emotional and social responses to abusive leadership. Secondly, the findings enrich the literature on core self-evaluation by demonstrating its dual role as a protective factor against emotional exhaustion and a facilitator of ingratiation behavior in the context of abusive supervision.

The study’s findings offer practical guidance for human resource practices and leadership development. Organizations should implement regular supervisor training programs that emphasize emotional intelligence and ethical behavior to reduce the prevalence of abusive supervision, both in business and public management (Li et al., 2022). Monitoring early signs of emotional exhaustion in employees can help prevent escalation into deviant behavior. Additionally, cultivating CSE through coaching or personal development programs may serve as a psychological buffer and promote constructive interpersonal coping strategies such as IB rather than reactive aggression.

Despite its contributions, this study has several limitations. First, data were self-reported and collected from a single source, which may introduce social desirability or common method bias, although CLF testing suggested minimal impact. Second, the study was conducted in the Chinese cultural context, where hierarchical leadership is prevalent. This may limit the generalizability of findings to other cultural or organizational environments. Third, our model focused on CSE as a moderator, but other factors such as organizational support or job control may also shape how employees respond to abusive supervision.

Future research could build on our findings in several ways. First, longitudinal or experimental designs could provide stronger causal evidence of the psychological mechanisms observed. Second, future studies may include multi-source or peer-reported data to reduce bias. Third, expanding the model to include other regulatory traits or contextual variables (e.g., organizational justice, climate for voice) could provide deeper insights into the dynamics of abusive supervision.

Despite its contributions, this study is not without limitations. Firstly, the data was collected using self-report measures, which may introduce common method bias. Future research could benefit from incorporating multiple sources of data, such as supervisor ratings or objective performance metrics. Secondly, the study focused on employees under Chinese context, limiting the generalizability of the findings. Future studies should replicate these findings across diverse organizational contexts to ensure robustness and applicability across different settings.

### 4.2 Future directions

Building on the current findings, several avenues for future research emerge. Firstly, longitudinal studies could explore the temporal dynamics of abusive supervision, emotional exhaustion, ingratiation behavior, and core self-evaluation over time. This would provide a clearer understanding of how these variables unfold and interact over the course of employment relationships. Secondly, investigating additional mediators and moderators could further elucidate the complex mechanisms underlying the effects of abusive supervision. For instance, exploring the role of organizational culture or leadership interventions in mitigating the impacts of abusive supervision could offer additional insights into effective organizational practices. Finally, cross-cultural studies could examine whether these relationships vary across different cultural contexts, thereby enhancing the cultural sensitivity and applicability of the findings.

In conclusion, this study contributes nuanced insights into the relationships between abusive supervision, employee behaviors, and the moderating role of core self-evaluation. By unpacking the mediating pathways through emotional exhaustion and ingratiation behavior, as well as highlighting the moderating effects of core self-evaluation, this research enhances our understanding of how abusive supervision shapes employee responses and behaviors in organizational settings.

## Data Availability

The raw data supporting the conclusions of this article will be made available upon request for academic research. Requests to access the data should be directed to the corresponding author.
